# Different levels of autophagy induced by transient serum starvation regulate metabolism and differentiation of porcine skeletal muscle satellite cells

**DOI:** 10.1038/s41598-023-40350-y

**Published:** 2023-08-12

**Authors:** Yi Wang, Juan Gao, Bojun Fan, Yuemin Hu, Yuefei Yang, Yajie Wu, Feng Li, Huiming Ju

**Affiliations:** 1 College of Veterinary Medicine, Yangzhou University/Jiangsu Co-Innovation Center for Prevention and Control of Important Animal Infectious Diseases and Zoonosis, Yangzhou, 225009 Jiangsu People’s Republic of China; 2 Biocytogen JiangSu Co., Ltd., Nantong, 226000 Jiangsu People’s Republic of China; 3 Department of Reproductive Medicine Center, Northern Jiangsu People’s Hospital Affiliated to Yangzhou University/Clinical Medical College, Yangzhou University, Yangzhou, 225009 Jiangsu People’s Republic of China

**Keywords:** Developmental biology, Stem cells, Zoology

## Abstract

This study aimed to investigate the effects of different levels of autophagy induced by transient serum starvation on the metabolism, lipid metabolism, and differentiation of porcine skeletal muscle satellite cells (SMSCs) to preliminary elucidate the role and function of autophagy in the regulatory network of skeletal muscle development. Different levels of autophagy were induced by controlling the serum concentration in the culture system for 24 h. Apoptosis, membrane potential, reactive oxygen species (ROS), ATP, and myogenic and lipogenic differentiation markers were monitored to determine if autophagy affected the metabolism and differentiation of SMSCs. Autophagy was induced in SMSCs via serum starvation (5%, 15%), as evidenced by decreased p62 and mTOR phosphorylation levels and increased LC3B lipidation and AMPK phosphorylation levels. Transmission electron microscopy revealed the presence of autophagosomes, and the rates of morphologically abnormal nuclei and mitochondria gradually increased with the decrease in serum concentration, the number of autophagic lysosomes also increased, indicating that 5% serum starvation induced severe autophagy, while 15% serum starvation induced mild autophagy. Compared with the control group and 15% serum-starved SMSCs, SMSCs undergoing 5% serum starvation had the highest intracellular ATP and ROS levels, the highest percentage of apoptotic cells, and the lowest membrane potential. The 15% serum-starved SMSCs had the highest membrane potential, but the percentage of apoptotic cells did not change significantly compared with the control group. The levels of the myogenic markers MyoD1 and MHC were significantly higher in 15% serum-starved SMSCs than in serum-sufficient SMSCs and the lowest in the 5% serum-starved SMSCs. The lipid contents (measured by Oil Red O staining and quantification of triglycerides) and lipogenic markers Peroxisome Proliferators-activated Receptors γ and Lipoprotein Lipase were also significantly higher in SMSCs undergoing 15% serum starvation than in the control group, and the lowest in the 5% serum-starved SMSCs. Different levels of starvation stress induce different levels of autophagy. Mild autophagy induced by moderate serum starvation promotes the metabolism and differentiation of SMSCs, while severe autophagy renders SMSCs more apoptotic, abnormal metabolism and suppresses SMSC differentiation into adipocytes or myocytes, and reduces lipid metabolisms. Our study suggests that autophagy plays a role in skeletal muscle development and may help design strategies for improving meat production traits in domestic pigs.

## Introduction

This study aimed to elucidate the role and function of autophagy in the regulatory network of skeletal muscle development and to provide a theoretical basis for the improvement of meat production traits in domestic pigs. The molecular mechanism underlying the growth and development of porcine skeletal muscles and the formation of meat production traits has always been the focus of attention in the field of animal genetics and breeding. Although some major breakthroughs and progress have been made in recent years, there are still many unknown regulatory factors and mechanisms to be studied. Skeletal muscle satellite cells (SMSCs), which are adult stem cells residing in the skeletal muscle tissue in an undifferentiated and quiescent state^1^, were first discovered in 1961. When muscle growth is damaged or stressed due to muscle degenerative diseases, satellite cells can form muscle fibers through myogenic differentiation^2^. The key transcription factors involved in this process include Myogenic Differentiation (MyoD) and Myosin Heavy Chain (MHC). SMSCs are multi-functional stem cells, which can differentiate into both muscle cells and adipocytes^3,4^. In the process of adipocyte development and formation, Peroxisome Proliferators-activated Receptors γ (PPARγ) and Lipoprotein Lipase (LPL) are considered to be the main regulatory factors that can promote the differentiation and formation of adipocytes^5,6^. During cell culture, appropriate concentrations of serum can provide a wide range of proteins, amino acids, and growth factors required for cell growth. Changes in intracytoplasmic amino acid concentrations caused by starvation and increases in intracellular AMP/ATP ratios caused by nutrient deficiency can lead to the activation of AMPK and precise inhibition of mTORC1 activity, thereby inducing autophagy^7^. Autophagy—the formation of autophagosomes that shuttle macromolecules, including proteins and organelles, such as mitochondria, into lysosomes for degradation into amino acids and monosaccharides^8^—modulates the recycling and reuse of energy and matter and is a highly conserved process^9^. Studies have shown that the autophagy fluxes increase in skeletal muscles under starvation, disuse atrophy, hypoxia, and lack of exercise^10–12^. Multiple factors and signaling pathways have been demonstrated to be involved in the regulation of autophagy flux. Among them, reactive oxygen species (ROS) are involved in the control of autophagy flux and are directly related to the regeneration and repair of skeletal muscles. A certain amount of ROS is essential for regulating cell growth and maintaining various biological functions of cells, but some degenerative diseases, skeletal muscle diseases, diabetes, and aging can lead to the destruction of the ROS balance, thus affecting skeletal muscle metabolism^13^. Although there is evidence that the muscle recovery process is closely related to autophagy, the specific pathways and precise mechanisms by which autophagy affects muscle regeneration and repair remain to be systematically studied^10,14^. In this study, we induced autophagy of varying degrees in a serum-containing SMSC culture system and observed the effects of different degrees of autophagy on cell metabolism and differentiation functions. The results of our study not only help to explore the factors affecting skeletal muscle development after pig birth but also lay a foundation for understanding the mechanisms of skeletal muscle development in pigs.

## Materials and methods

### Cell grouping and treatment

Porcine SMSCs (cell lines were pre preserved and stored by our laboratory) were seeded in 6-well plates and divided into six groups for further culturing when the cells reached 50–60% confluence. The cells grown in culture medium containing 20% serum (FBS, Ref#10270-106, Gibco, Brazil) constituted the normal control group (20% S group). The cells grown in culture medium containing 15% serum were the 15% S group, and the cells grown in culture medium containing 5% serum were the 5% S group. Cells were also divided into groups and subjected to autophagy inhibition via treatment with 5 mM of the autophagy inhibitor 3-methyladenine (3-MA, HY-19312, MCE, USA). The groups were named as follows: 20% S + 3-MA, 15% S + 3-MA, and 5% S + 3-MA groups. After 24 h of culture under the corresponding conditions, cell metabolic indices and differentiation abilities of the cells were detected. The experiments were conducted in triplicate and repeated three times with similar results.

### Effects of different levels of autophagy on cell metabolism

#### Detection of autophagy-related protein expression by WB

After culturing the above six groups of cells for 24 h, the total protein of each was extracted. After polyacrylamide gel electrophoresis and membrane transfer, tubulin (11224-1-AP, Proteintech, USA) was used as the internal reference protein to detect the expression of autophagy-related proteins LC3B (ab229327, Abcam, USA), p62 (ab233207, Abcam, USA), AMPK (66536-1-lg, Proteintech, USA), phospho-AMPK (p-AMPK, GTX03702, GeneTex, USA), mTOR (66888-1-lg, Proteintech, USA), and phospho-mTOR (p-mTOR, 67778-1-lg, Proteintech, USA). MHC (sc-53088, Santa, USA) and MyoD1 (sc-32758, Santa, USA) antibodies were used to detect myogenesis. The gray value of the western blot (WB) hybridization band was measured by ImageJ software (National Institutes of Health, Bethesda, MD, USA). The ratio of the gray value of the target protein and the corresponding internal reference band was the relative expression of each target band. The relative expression of the proteins of interest was quantified and calculated as previously reported^15^.

#### Detection of mitochondria and autophagosome morphology changes by transmission electron microscopy

The cells of each group were fixed in 2.5% formaldehyde for 10 h at 4 °C, washed four times with 0.05 M phosphate buffer solution, fixed with 1% citric acid for 1.5 h, and dehydrated with gradient concentration ethanol. The samples were cut into slices (70 nm) and then stained with uranyl acetate and lead citrate. The morphological structure, number, and structure of mitochondria and autophagosomes in the cells were observed by transmission electron microscopy. The specific experiments were carried out as previously reported^16^.

#### Detection of lysosomes in cells

The cells of each group were inoculated into 24-well plates with 1 mL of Mito-Tracker (C1048, Beyotime, China) working solution and incubated for 30 min at 37 °C in a 5% CO_2_ incubator for mitochondrial staining; 1 mL of Lyso-Tracker (C1046, Beyotime, China) working solution was added to the cells before being incubated for 30 min at 37 °C in a 5% CO_2_ incubator. The cells were washed, observed using fluorescent microscopes, and photographed. The fluorescence intensity was analyzed by ImageJ software.

#### Detection of cell apoptosis rate, mitochondrial membrane potential, and ROS level by flow cytometry

After washing with PBS, the cells were digested with trypsin containing EDTA to prepare the cell suspension, and then, 195 µL of Annexin V-FITC, 5 µL of Annexin V-FITC, and 10 µL of iodinated propidium were added to the mixed solution. After incubating the mixture at 15 °C without light for 15 min, 200 µL of PBS was added, and the sample was filtered with a 100-mesh sieve. The apoptosis rate of each group of cells was detected by flow cytometry with the apoptosis reagent kit (C1062S, Beyotime, China). According to the instructions of the mitochondrial membrane potential reagent kit (C2006, Beyotime, China), JC-1 staining was used to detect the mitochondrial membrane potential of each group of cells by flow cytometry. The mitochondrial active oxygen (ROS) level in each group of cells was detected with the active oxygen detection reagent kit (S0033S, Beyotime, China).

#### Detection of intracellular ATP and related kinase levels

After washing with PBS, the cells were digested with trypsin containing EDTA to prepare the cell suspension. According to the instructions of the ATP detection kit (S0026, Beyotime, China), 200 µL of lysis solution was added to each well, and the sample was centrifuged at 12000 g for 5 min at 4 °C. The supernatant was discarded, and then, 100 µL of ATP detection working solution was added for 3–5 min, and the relative light units (RLUs) were obtained by reading the samples in a chemiluminescence plate reader. The ATP concentration in the sample was calculated according to the prepared standard curve. After washing with PBS, the cells were digested with trypsin containing EDTA, homogenized with saline (0.9% NaCl) at a volume ratio of 5:1, and centrifuged at 10000 g for 5 min at 4 °C. The supernatant was extracted, and the ATPase activity was measured using an ATPase Activity Assay Kit (E-BC-K831-M, Elabscience, China). The cells digested with trypsin containing EDTA were used to prepare the suspension, which was homogenized and then broken by ultrasound. Mitochondrial Complex V Activity Assay Kit (E-BC-K153-M, Elabscience, China) was used to determine ATP synthetase activity in the supernatant.

### Effect of different levels of autophagy on cell differentiation

#### Myogenesis and lipogenesis induction

After culturing the six groups of cells described in “Cell grouping and treatment” section for 24 h, myogenesis and lipogenesis were studied. After induction of myogenesis with myogenesis differentiation medium (2% HS horse serum + 1% double antibody + DMEM high sugar medium) for 4 days, the cells were collected for subsequent experiments. With lipogenesis induction solution (10% FBS + 5 μg mL^−1^ insulin + 1 μmol L^−1^ DEX + 0.5 mmol L^−1^ IBMX + 1 μmol L^−1^ rosiglitazone + 1% double antibody + DMEM high sugar medium) induction for 72 h, the cells were then changed to lipogenesis induction maintenance solution (10% FBS + 5 μg mL^−1^ insulin + 1% double antibody + DMEM high sugar medium) to maintain the lipogenesis induction.

#### Detection of myogenic differentiation markers by cellular immunofluorescence

The cells were fixed, permeabilized, and blocked. MHC and MyoD1 antibodies were incubated with the samples at 4 °C overnight, and then, the FITC fluorescent secondary antibody (FITC-Goat anti mouse IgG, 18943-1-AP, Proteintech, China) was incubated at room temperature for 2 h. DAPI was used to stain the cell nucleus for 10 min. MHC and MyoD1 protein expression was detected by a laser confocal microscope (TCS SP8 STED, Leica, USA). The experiment was repeated three times, and the fluorescence intensity was analyzed using ImageJ software for each group of samples.

#### Detection of adipogenic differentiation markers by qRT-PCR

Total mRNA was isolated from cells that went lipogenesis induction in each group and reverse transcribed into cDNA. The GAPDH gene was used as the internal reference, and qRT-PCR was used to detect the expression level of PPARγ and LPL mRNA in each group of cells. Quantitative addition and reaction systems were operated according to TB Green® Premix Ex Taq™ II (RR82LR, Takara, Japan) operation procedure, and the related primers were as follows, PPARγ F 5ʹ-AGAGTATGCCAAGAACATCC-3ʹ, R 5ʹ-ATCTAATTCCAGTGCGTTGA-3ʹ; LPL F 5ʹ-CAGAGGTGGATATTGGAGAG-3ʹ, R 5ʹ-GAGACTTGTCGTGGCATT-3ʹ; GAPDH F 5ʹ-AGCAATGCCTCCTGTACCAC-3ʹ, R 5ʹ-AAGCAGGGATGATGTTCTGG-3ʹ. The relative expression of the proteins of interest was quantified and calculated as previously reported^17^.

#### Detection of fat content by Oil Red O

After lipogenesis induction and rinsing of each group of cells, the newly prepared Oil Red O fixative was incubated with the cells for 15–20 min. Then, the cells were rinsed with distilled water and then with 60% isopropanol for 5 min. Then, 1 mL of the Oil Red O staining solution (G1262, Solarbio, China) was added, and the cells were stained at 37 °C for 30–40 min. The staining solution was discarded, and the cells were rinsed with distilled water. The results were observed under a microscope. Then, 1 mL of isopropanol was added to each well of a six-well plate to extract the fat droplets and incubated at 37 °C on a constant temperature shaker for 15 min. The Oil Red O quantitative optical density (OD) value was detected by a plate reader at 510 nm.

#### Detection of TG levels in cells

After lipogenesis induction, the cells were digested with pancreatin, centrifuged at 800 rpm for 10 min, and then the supernatant was discarded. Equal ratios of isopropanol and n-hexane were added, followed by ultrasonic lysing for 1 min and centrifugation at 4 °C at 8000×*g* for 10 min. The supernatant was collected as the triglyceride (TG) test solution. The operation was performed according to the TG detection kit (BC0620, Solarbio, China), and the absorbance value at 420 nm was detected by a plate reader. According to the statistical number of cells, the TG content in each group of cells was quantitatively calculated.

### Statistical analysis

Data were presented as the x ± SD. All statistical analyses were performed using GraphPad Prism7 software (GraphPad Software, LA Jolla, CA, USA). Generally, the cytokine mRNA levels, protein expression levels were analyzed using two-tailed Student’s t-tests, and differences were considered statistically significant when *P < 0.05 and **P < 0.01.

## Results

### Effect of different levels of autophagy on cell metabolism

#### Detection of autophagy-related protein expression by WB

**Figure 1:**
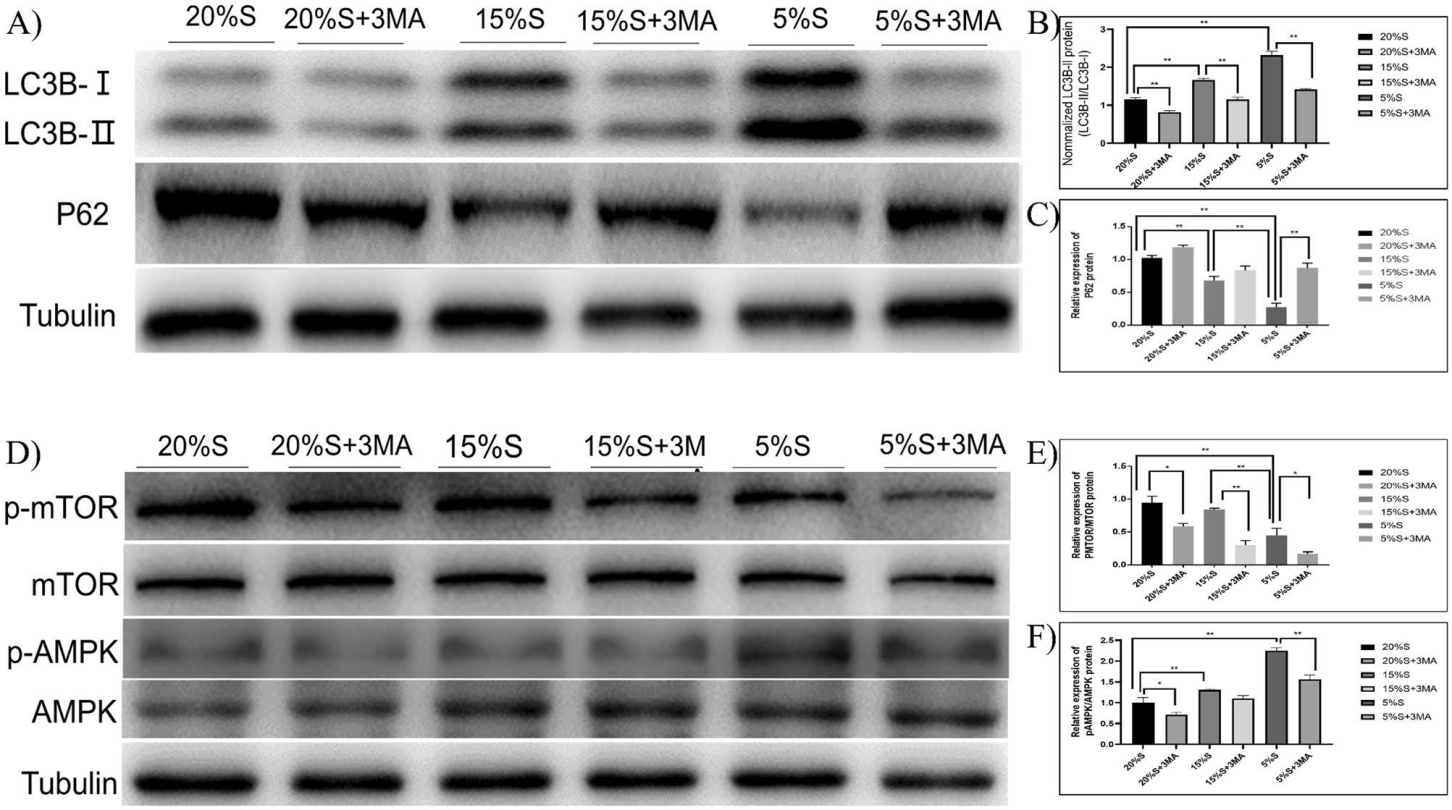
Detection of autophagy-related protein expression by WB. (**A**) WB results of LC3B-II and P62; (**B**) Grayscale analysis of LC3B-II/LC3B-I; (**C**) Grayscale analysis of P62; (**D**) WB results of autophagy signal pathway proteins AMPK, p-AMPK, mTOR and p-mTOR; (**E**) Grayscale analysis of P-mTOR/mTOR; (**F**) Grayscale analysis of p-AMPK/AMPK. The gel images of mTOR and AMPK were cropped from the same membrane. The blots were cut prior to hybridisation with antibodies. The data are expressed as the mean ± SD (n = 3) of three experiments. The statistical significance of differences between groups was analyzed using two-tailed Student’s t-tests, and differences were considered statistically significant when *P < 0.05 and **P < 0.01 (Supplementary information 1).

Using tubulin as the internal reference, WB was used to detect the expression of autophagy marker proteins LC3B, p62, AMPK, p-AMPK, mTOR, and p-mTOR. The detection results are shown in Fig. 1. In the 20% S, 20% S + 3-MA, 15% S, 15% S + 3-MA, 5% S, and 5% S + 3-MA groups of cells, the ratios of grayscale values of LC3B-II/LC3B-I hybridization strips were 1.15 ± 0.05, 0.81 ± 0.04, 1.66 ± 0.05, 1.15 ± 0.06, 2.32 ± 0.1, and 1.41 ± 0.03, respectively; the relative expression of p62 protein was 1.0212 ± 0.058, 1.1895 ± 0.041, 0.6778 ± 0.091, 0.8322 ± 0.097, 0.2737 ± 0.086, and 0.8702 ± 0.105, respectively; the expression of p-mTOR/mTOR was 0.9458 ± 0.099, 0.5883 ± 0.041, 0.8382 ± 0.023, 0.3031 ± 0.068, 0.4481 ± 0.108, and 0.1684 ± 0.03, respectively; and the p-AMPK/AMPK ratios were 0.99 ± 0.08, 0.71 ± 0.03, 1.37 ± 1.38, 1.09 ± 1.1, 2.25 ± 0.04, and 1.56 ± 0.06, respectively. The above results showed that the degree of autophagy in the 20% S group was significantly lower than that of the 15% S group, and the degree of autophagy in the 5% S group was the highest (*P* < 0.01). Compared with the corresponding experimental group, the degree of autophagy in the 3-MA groups was significantly reduced (*P* < 0.05).

#### Detection of mitochondria and autophagosome morphology changes by transmission electron microscopy

**Figure 2:**
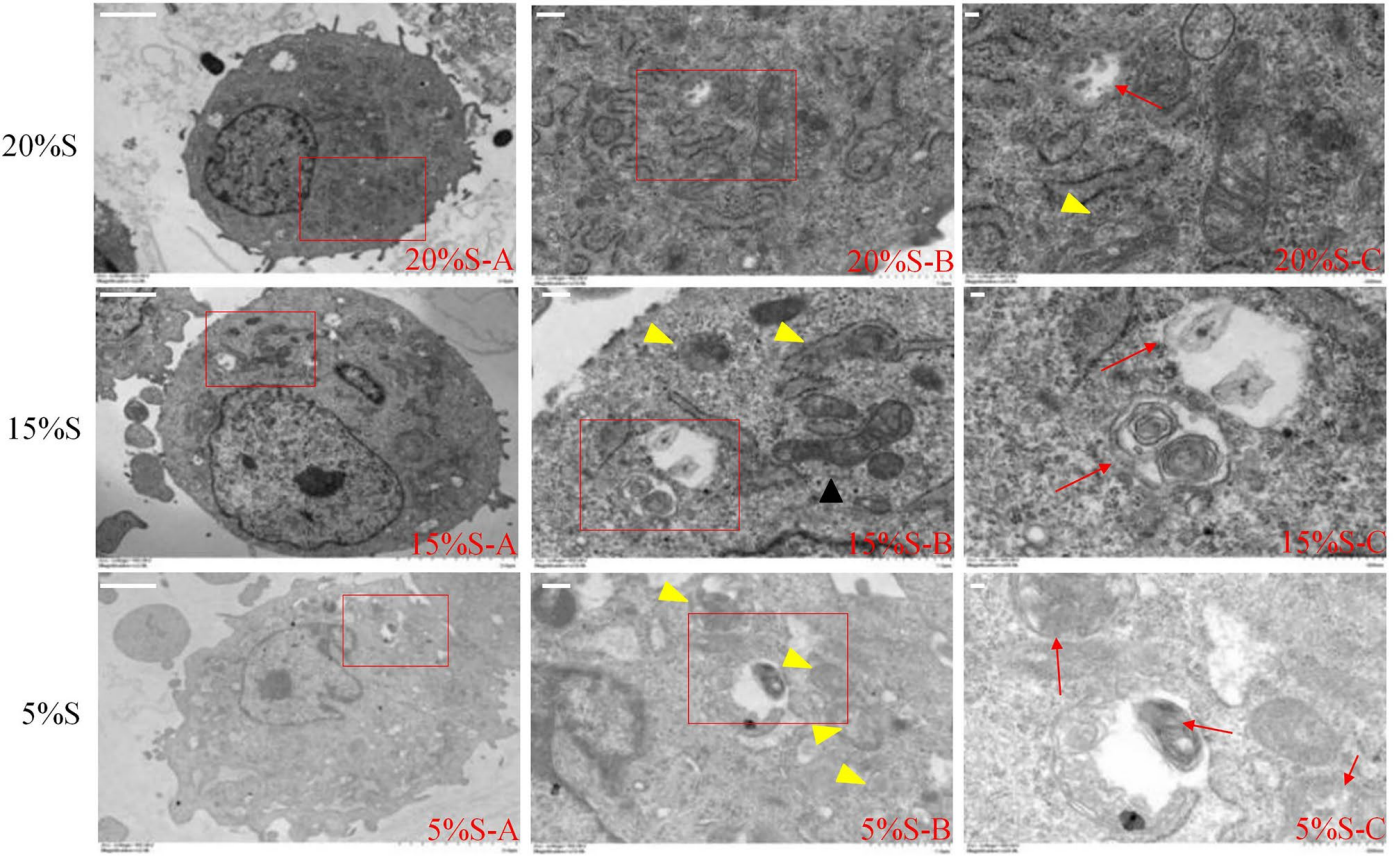
Detection of mitochondria and autophagosome morphology changes by transmission electron microscopy. With the decrease in serum concentration, the number of autophagic lysosomes (as shown by the red arrow), the proportion of mitochondrial swelling and blurring of mitochondrial cristae, and the rate of abnormal nucleus and mitochondria (as shown by the yellow triangle) all increases. Scale: (**A**) 2.0 µm, (**B**) 1.0 µm, (**C**) 500 nm.

Morphological changes in the mitochondria and autophagosomes in cells were detected by transmission electron microscopy (Fig. 2). In the 20% S group, the cell structure was normal, mitochondria were abundant, the morphology and structure of mitochondria were normal, the nuclear cell membrane was intact, and a few autophagic bodies were visible in the cytoplasm. The number of autophagic bodies in the 15% S group was significantly higher than that in the 20% S group, and the proportion of mitochondrial swelling and blurred mitochondrial cristae also significantly increased. In the 5% S group, most of the mitochondria were swollen, the mitochondria cristae disappeared, the nuclei were heterotypic, and the number of autophagic bodies in the cells was the highest.

#### Detection of lysosomes in cells

**Figure 3:**
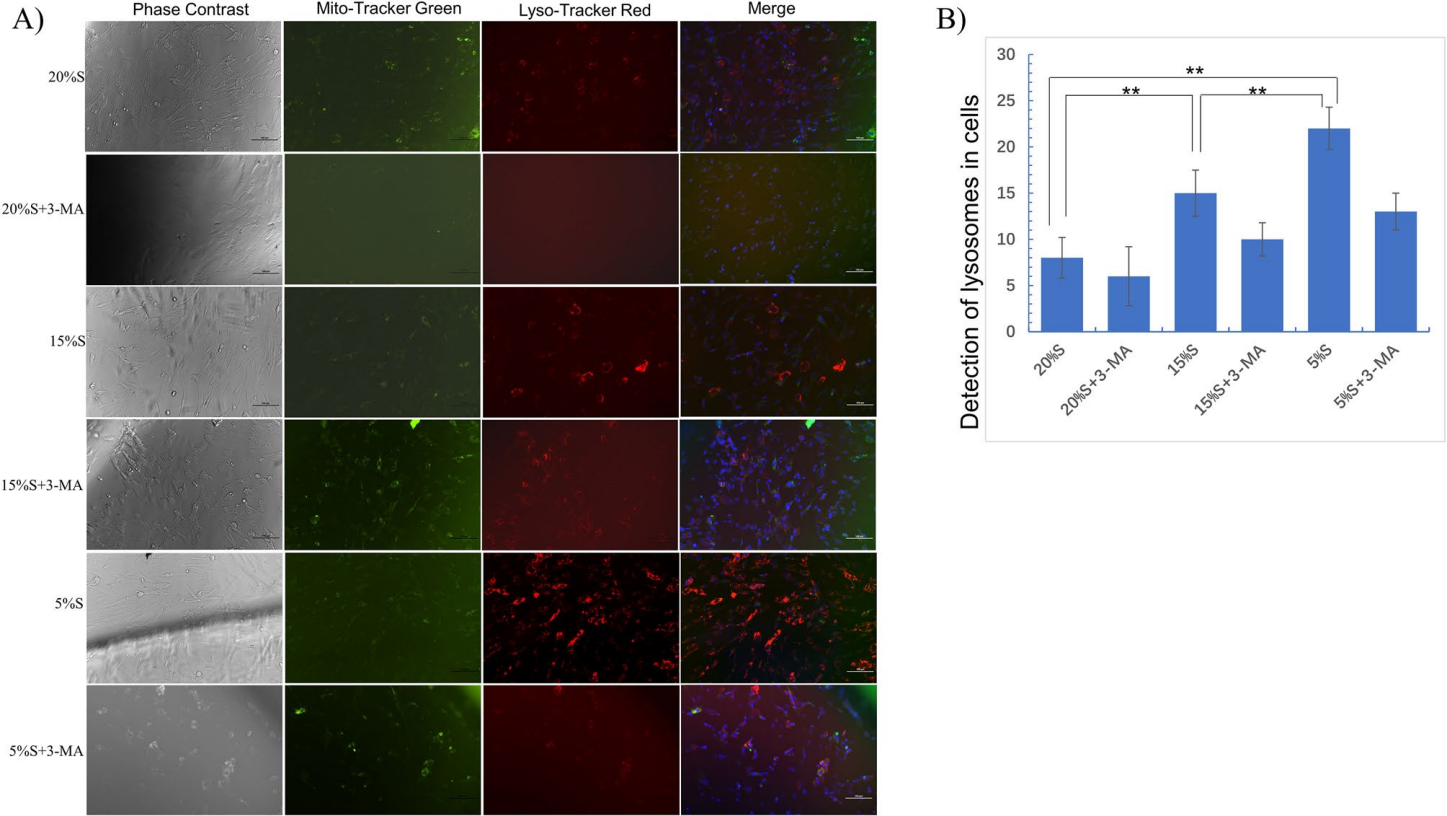
Detection of lysosomes in cells. (**A**) Mito-Tracker Green staining of intracytoplasmic mitochondria with green signal; Lyso-Tracker Red staining of cytosolic lysosomes with red fluorescence signal and superimposition of red fluorescence and blue fluorescence in the nucleus (merge). The data are expressed as the mean ± SD (n = 3) of three experiments. The statistical significance of differences between groups was analyzed using two-tailed Student’s t-tests, and differences were considered statistically significant when *P < 0.05 and **P < 0.01.

After labeling mitochondria and lysosomes with Mito-Tracker and Lyso-Tracker, respectively, the results were observed using fluorescence microscopy (Fig. 3). The number of autophagic lysosomes was significantly higher in the 15% S group than in the 20% S group, and the highest number was found in the 5% S group. Compared with the corresponding experimental group, the number of lysosomes in the 3-MA groups was significantly reduced (*P* < 0.05). This indicated that autophagy was more severe in the 15% S and 5% S groups than in the control group, especially in the 5% group.

#### Detection of apoptosis rate by flow cytometry

**Figure 4:**
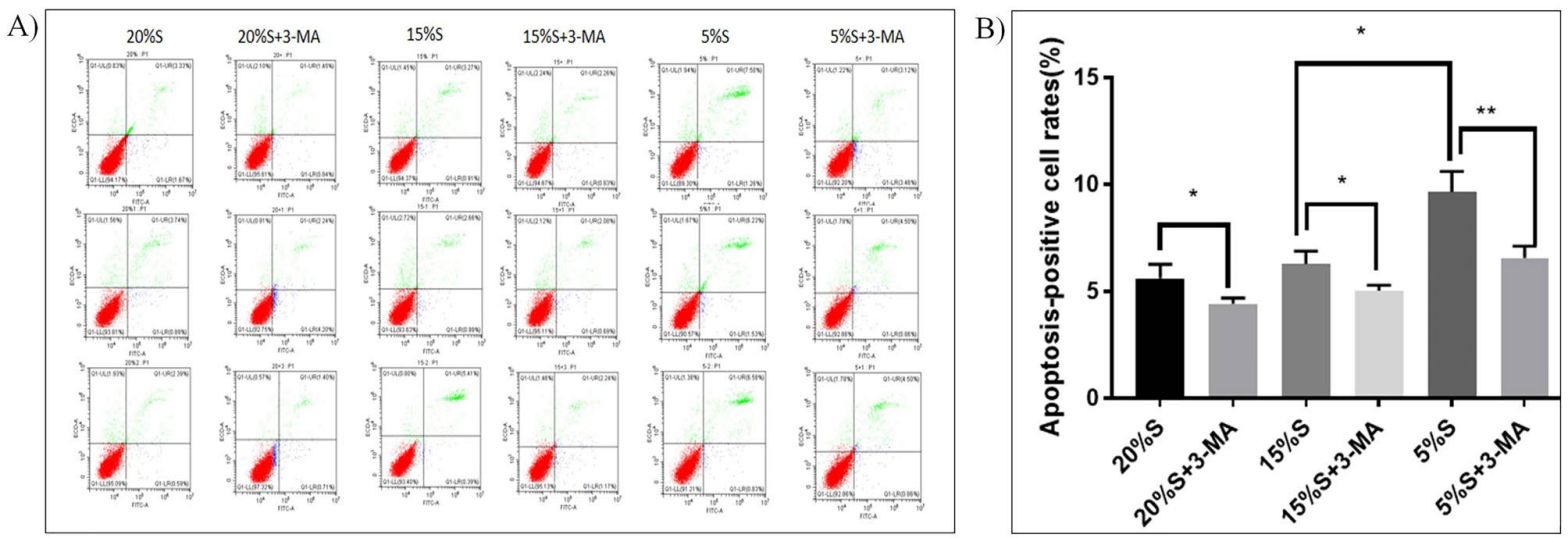
Detection and analysis of apoptosis rate. (**A**) Detection of apoptosis rate by flow cytometry. The Q1 quadrant is mechanical injury. The Q2 quadrant is early apoptosis. The Q3 quadrant is late apoptosis, and the Q4 quadrant is normal cells. (**B**) Analysis of apoptosis rate. The data are expressed as the mean ± SD (n = 3) of three experiments. The statistical significance of differences between groups was analyzed using two-tailed Student’s t-tests, and differences were considered statistically significant when *P < 0.05 and **P < 0.01.

Flow cytometry was used to detect the apoptosis rate of the cells. The results are shown in Fig. 4. The apoptosis rates of cells of the 20% S, 20% S + 3-MA, 15% S, 15% S + 3-MA, 5% S, and 5% S + 3-MA groups were 5.62 ± 0.52, 4.44 ± 0.21, 6.27 ± 0.49, 5.03 ± 0.21, 9.64 ± 0.79, and 6.55 ± 0.46, respectively. The apoptosis rate of the 5% S group was significantly higher than that of the 15% S group and 20% S group (*P* < 0.05). There was no significant difference between the 15% S group and 20% S group. Compared with the corresponding experimental group, the cell apoptosis rate was significantly inhibited in the 3-MA groups (*P* < 0.05).

#### Detection of mitochondrial membrane potential by flow cytometry

**Figure 5:**
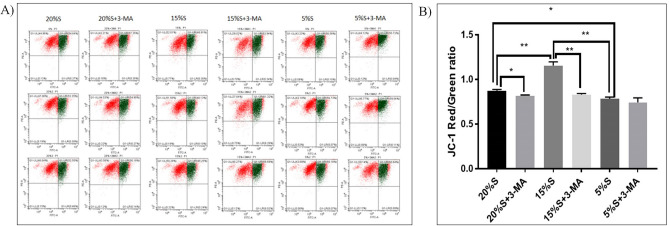
Detection and analysis of membrane potential. (**A**) Detection of the mitochondrial membrane potential by flow cytometry; (**B**) Analysis of membrane potential by JC-1 red-green fluorescence ratio. The data are expressed as the mean ± SD (n = 3) of three experiments. The statistical significance of differences between groups was analyzed using two-tailed Student’s t-tests, and differences were considered statistically significant when *P < 0.05 and **P < 0.01.

The mitochondrial membrane potential in the cells of each group was detected by flow cytometry. The results are shown in Fig. 5. The red-green fluorescence ratio of the cells of the 20% S, 20% S + 3-MA, 15% S, 15% S + 3-MA, 5% S, and 5% S + 3-MA groups was 0.87 ± 0.01, 0.82 ± 0.01, 1.12 ± 0.07, 0.83 ± 0.01, 0.78 ± 0.02, and 0.85 ± 0.08, respectively. The results showed that the mitochondrial membrane potential of the 15% S group was 0.25 ± 0.06 and 0.34 ± 0.05 higher than that of the 20% S group and 5% S group, respectively, with extremely significant differences (*P* < 0.01). After adding 3-MA to the 20% S group and 15% S group, the mitochondrial membrane potential decreased by 0.05 ± 0.01 and 0.29 ± 0.06, respectively, with a significant difference (*P* < 0.05).

#### Detection of mitochondrial ROS level by flow cytometry

**Figure 6:**
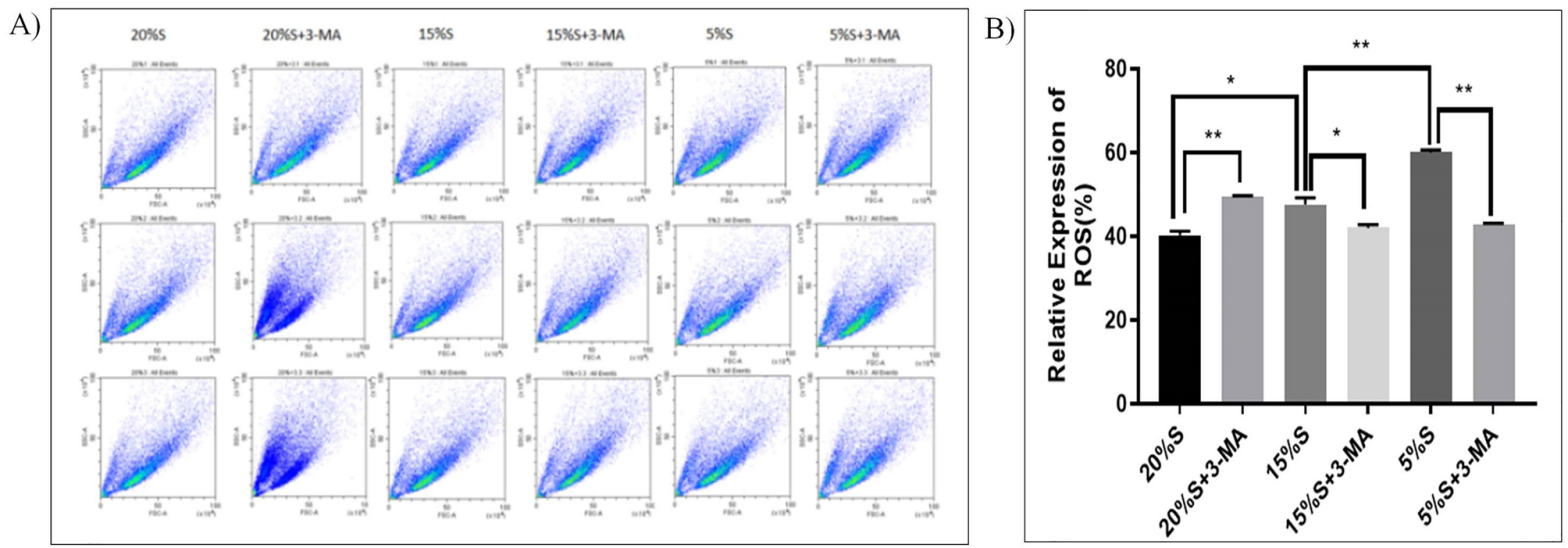
Detection and analysis of mitochondrial ROS levels. (**A**) Detection of ROS levels by flow cytometry; (**B**) Analysis of ROS levels. The data are expressed as the mean ± SD (n = 3) of three experiments. The statistical significance of differences between groups was analyzed using two-tailed Student’s t-tests, and differences were considered statistically significant when *P < 0.05 and **P < 0.01.

The ROS levels in the cells of each group were detected by flow cytometry. The results are shown in Fig. 6. The ROS levels in the cells of the 20% S, 20% S + 3-MA, 15% S, 15% S + 3-MA, 5% S, and 5% S + 3-MA groups were 40.26 ± 0.93, 49.47 ± 0.06, 47.54 ± 2.55, 42.01 ± 0.62, 60.06 ± 0.31, and 42.67 ± 0.19, respectively. The ROS level in the 5% S group was significantly higher than that in the 15% S group, while the ROS levels in the 15% S group and 5% S group were significantly higher than that in the 20% S group (*P* < 0.05). The ROS level in the 20% S + 3-MA group was significantly higher than that in the 20% S group, while the ROS levels in the 15% S + 3MA group and 5% S + 3-MA group were significantly lower than those in the 15% S group and 5% S group (*P* < 0.05).

#### Detection of intracellular ATP and related kinase levels

**Figure 7:**
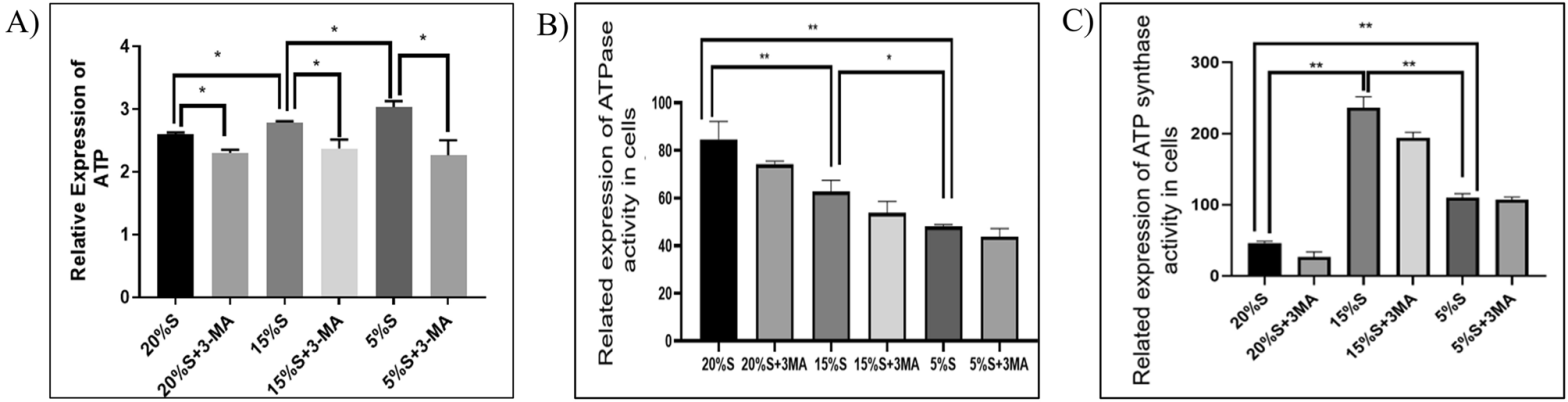
Detection and analysis of intracellular ATP and related kinase levels. (**A**) Intracellular ATP level analysis; (**B**) Intracellular ATPase level analysis; (**C**) Intracellular ATP synthase level analysis. The data are expressed as the mean ± SD (n = 3) of three experiments. The statistical significance of differences between groups was analyzed using two-tailed Student’s t-tests, and differences were considered statistically significant when *P < 0.05 and **P < 0.01.

The ATP levels in the cells in each group were detected with the ATP detection kit. The quantitative values of ATP in the 20% S, 20% S + 3-MA, 15% S, 15% S + 3-MA, 5% S, and 5% S + 3-MA groups were 2.6 ± 0.03, 2.3 ± 0.04, 2.78 ± 0.02, 2.37 ± 0.10, 3.03 ± 0.08, and 2.26 ± 0.17, respectively. The value of ATP in the 15% S group was significantly higher than that in the 20% S group (*P* < 0.05), while the value of ATP in the 5% S group was significantly higher than those in the 15% S and 20% S groups (*P* < 0.05). Compared with the corresponding experimental group, the ATP levels in the 3-MA groups were significantly reduced (*P* < 0.05) (Fig. 7).

ATPase and ATP synthase were detected with the corresponding Kit. The quantitative values of ATPase in the 20% S, 20% S + 3-MA, 15% S, 15% S + 3-MA, 5% S, and 5% S + 3-MA groups were 74.16 ± 1.42, 62.75 ± 4.75, 53.76 ± 4.83, 48.08 ± 0.79, 43.9 ± 3.26,41.9 ± 4.26, respectively; the ATP synthase values were 46.26 ± 2.69, 26.85 ± 7.12, 236.39 ± 15.34, 194.29 ± 7.6, 110.24 ± 5.37, 107.37 ± 3.52, respectively. The value of ATPase decreased with the decrease of serum concentration. The value of ATP synthase in the 15% S group were significantly higher than in other groups (*P* < 0.01). The value of ATPase in the 5% S group was the lowest (*P* < 0.01). (Fig. 7).

### Effect of different levels of autophagy on cell differentiation

#### Detection of myogenic differentiation markers by WB

**Figure 8:**
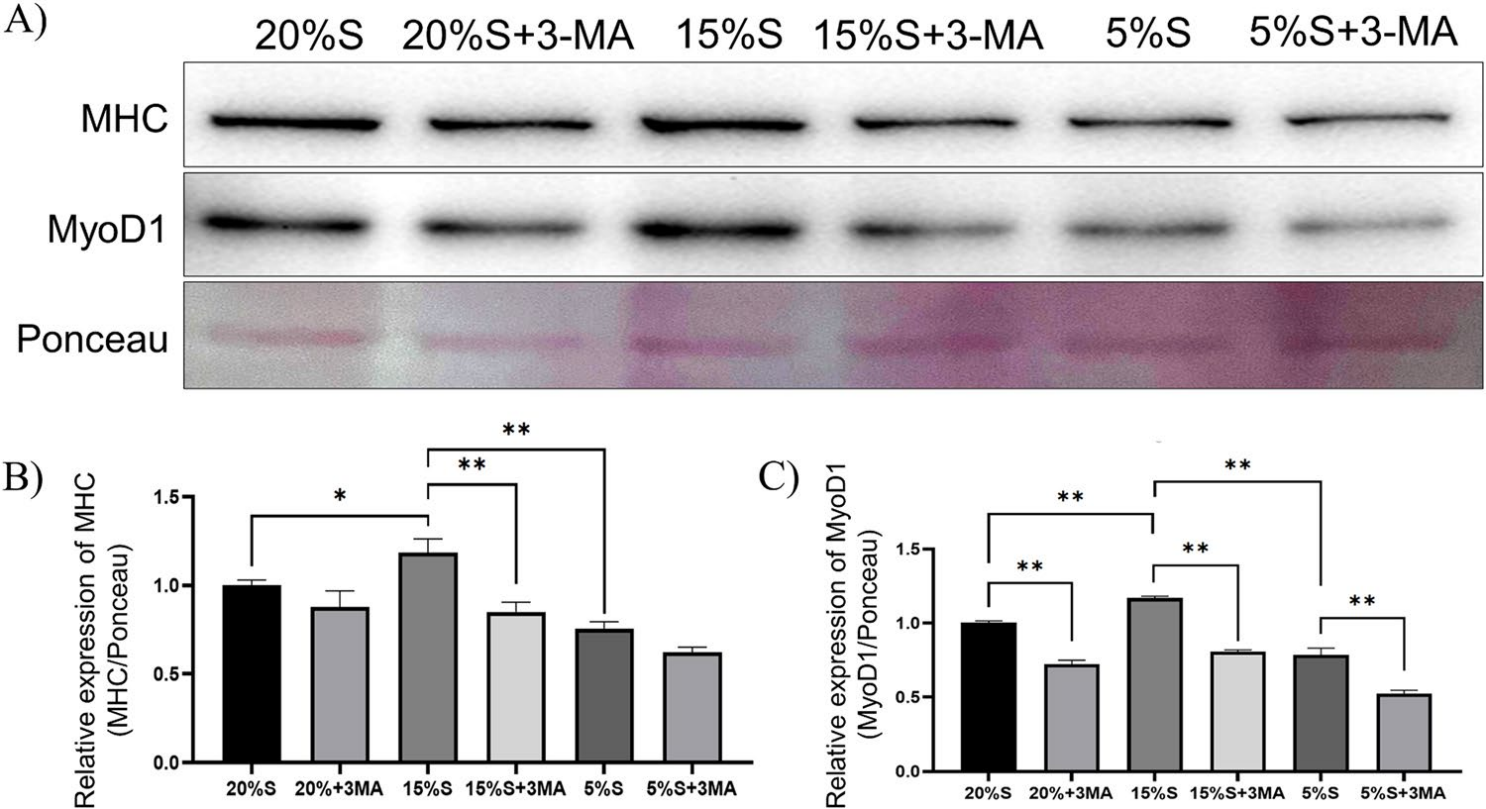
Detection and analysis of myogenic differentiation markers by WB. (**A**) WB results of MHC and MyoD; (**B**) Grayscale analysis of MHC; (**C**) Grayscale analysis of MyoD1. The data are expressed as the mean ± SD (n = 3) of three experiments. The statistical significance of differences between groups was analyzed using two-tailed Student’s t-tests, and differences were considered statistically significant when *P < 0.05 and **P < 0.01 (Supplementary information 2).

WB was used to detect the expression of myogenic differentiation marker proteins MHC and MyoD1. The results are shown in Fig. 8. The relative expression of MHC in the cell proteins of the 20% S, 20% S + 3-MA, 15% S, 15% S + 3-MA, 5% S, and 5% S + 3-MA groups was 1 ± 0.12, 0.83 ± 0.05, 1.31 ± 0.15, 0.9 ± 0.05, 0.59 ± 0.15, and 0.48 ± 0.12, respectively, and the relative expression of MyoD1 protein was 1 ± 0.18, 0.71 ± 0.05, 1.41 ± 0.15, 0.51 ± 0.09, 0.48 ± 0.09, and 0.32 ± 0.08, respectively. The expression of MHC and MyoD1 in the 15% S group was significantly higher than those in other groups (*P* < 0.01 or *P* < 0.05). After adding 3-MA, the relative expression of MyoD1 in the 15% S group and 5% S group decreased significantly, and no significant difference was observed in MHC expression among the three 3-MA groups.

#### Detection of myogenic differentiation markers by cellular immunofluorescence

**Figure 9:**
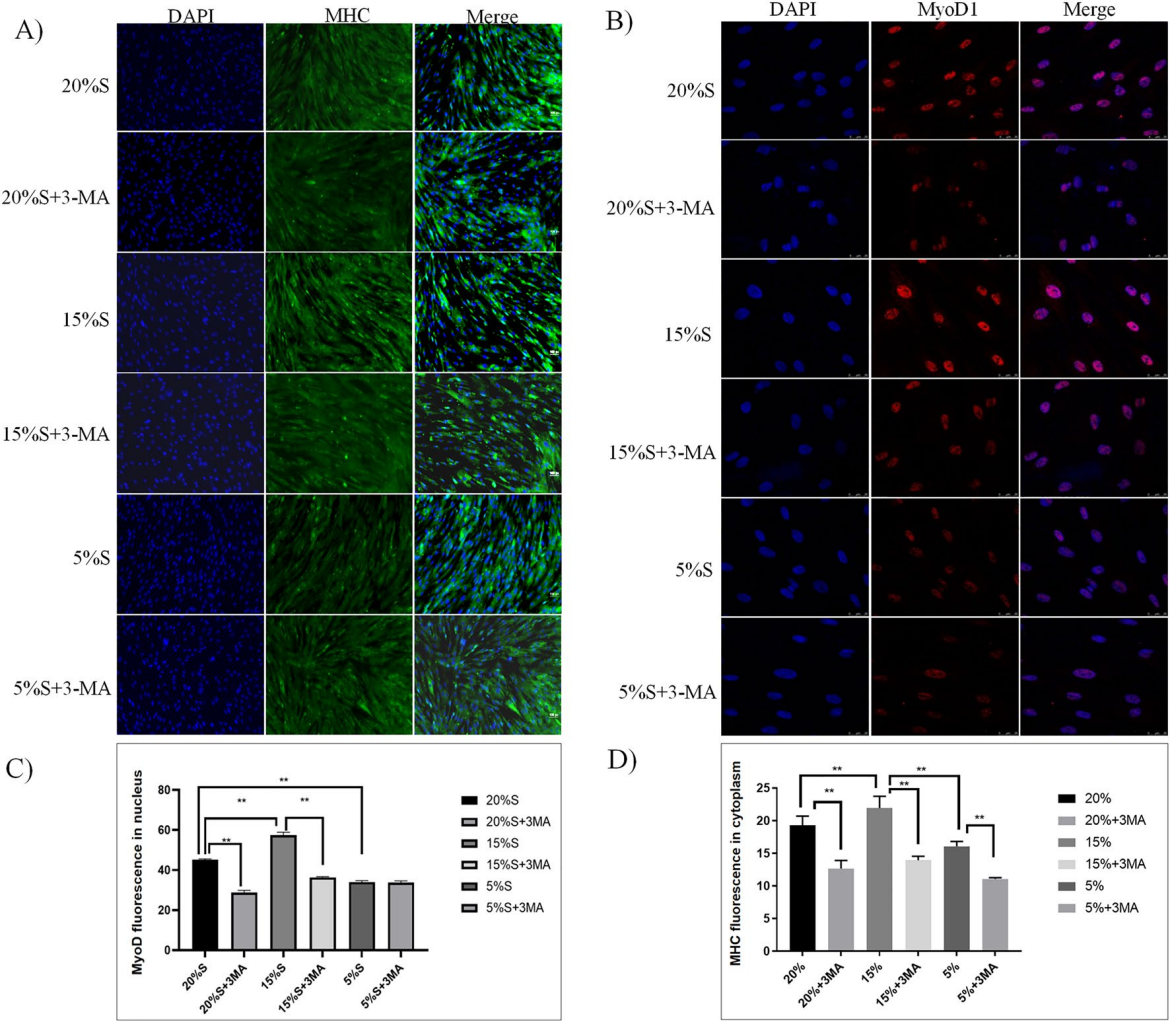
Detection and analysis of myogenic differentiation markers by cellular immunofluorescence. (**A**) Immunofluorescence staining results of MyoD1; (**B**) Immunofluorescence staining results of MHC; (**C**) Quantitative analysis of MyoD1 fluorescence; (**D**) Quantitative analysis of MHC fluorescence. The data are expressed as the mean ± SD (n = 3) of three experiments. The statistical significance of differences between groups was analyzed using two-tailed Student’s t-tests, and differences were considered statistically significant when *P < 0.05 and **P < 0.01.

Immunofluorescence was used to detect the expression of myogenic regulatory factors MyoD and MHC. The results are shown in Fig. 9. The expression of MyoD1 and MHC was detected in all groups after lipogenic differentiation. The average fluorescence values of cells in each group were quantitatively measured by ImageJ software. The fluorescence values of MyoD1 in the 20% S, 20% S + 3-MA, 15% S, 15% S + 3-MA, 5% S, and 5% S + 3-MA groups were 45.22 ± 1.17, 29.84 ± 1.76, 57.46 ± 1.46, 35.93 ± 0.60, 34.65 ± 1.49, and 34.68 ± 0.87, respectively, and MHC fluorescence values were 19.33 ± 1.36, 12.67 ± 1.23, 21.94 ± 1.79, 13.94 ± 0.60, 16.07 ± 0.76, and 11.07 ± 0.19, respectively. The expression of MoyD1 protein in the 15% S group was significantly higher than that in the 20% S group and 5% S group (*P* < 0.01). After adding 3-MA to inhibit autophagy, the expression of MoyD1 in the 20% S group and 15% S group decreased significantly, and there was no significant difference in the expression of MoyD1 among the three autophagy inhibition groups (*P* > 0.05). The change was basically consistent with the results of the WB detection of MoyD1 and MHC protein expression.

#### Detection of adipogenic differentiation markers by qRT-PCR

**Figure 10:**
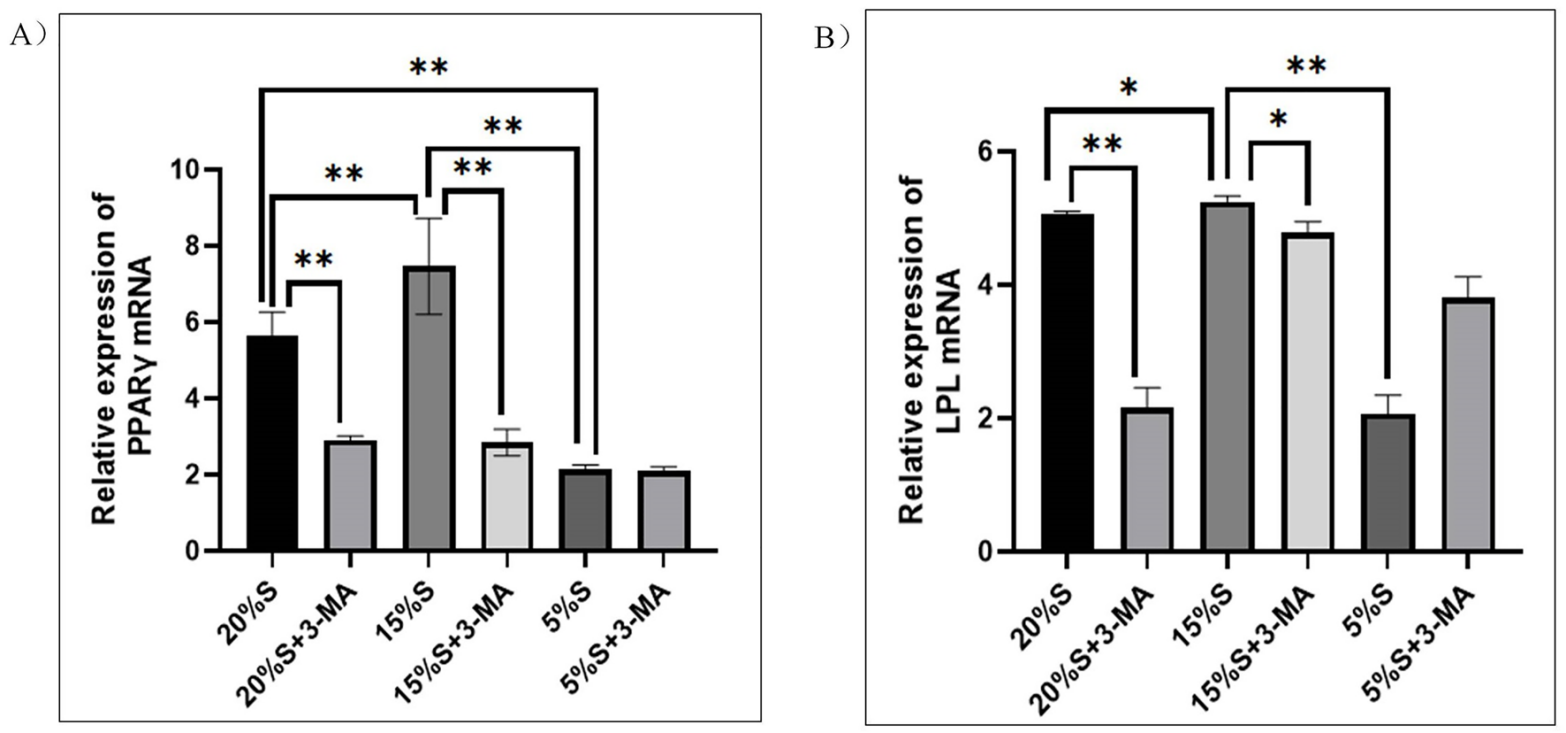
Detection and analysis of adipogenic differentiation markers by qRT-PCR. (**A**) Expression of PPARγ mRNA; (**B**) Expression of LPL mRNA. The data are expressed as the mean ± SD (n = 3) of three experiments. The statistical significance of differences between groups was analyzed using two-tailed Student’s t-tests, and differences were considered statistically significant when *P < 0.05 and **P < 0.01.

After adipogenic differentiation, PPARγ and LPL expression related to adipogenic differentiation was detected by qRT-PCR. The results are shown in Fig. 10. The mRNA expression level of PPARγ gene in the 20% S, 20% S + 3-MA, 15% S, 15% S + 3-MA, 5% S, and 5% S + 3-MA groups was 5.63 ± 0.52, 2.87 ± 0.06, 7.46 ± 1.03, 2.84 ± 0.34, 2.18 ± 0.07, and 2.10 ± 0.08, respectively, and the mRNA expression level of the LPL gene was 5.06 ± 0.03, 2.37 ± 0.43, 5.25 ± 0.07, 4.85 ± 0.18, 2.07 ± 0.23, and 3.81 ± 0.25, respectively. The expression level of PPARγ and LPL genes in the 15% S group was significantly higher than that of the 20% S group, 5% S group, and three inhibitor groups (*P* < 0.05 or *P* < 0.01). The relative expression level of PPAR γ and LPL genes decreased significantly in the 20% S + 3-MA group and the 15% S + 3-MA group (*P* < 0.05 or *P* < 0.01).

#### Detection of fat content by Oil Red O

**Figure 11:**
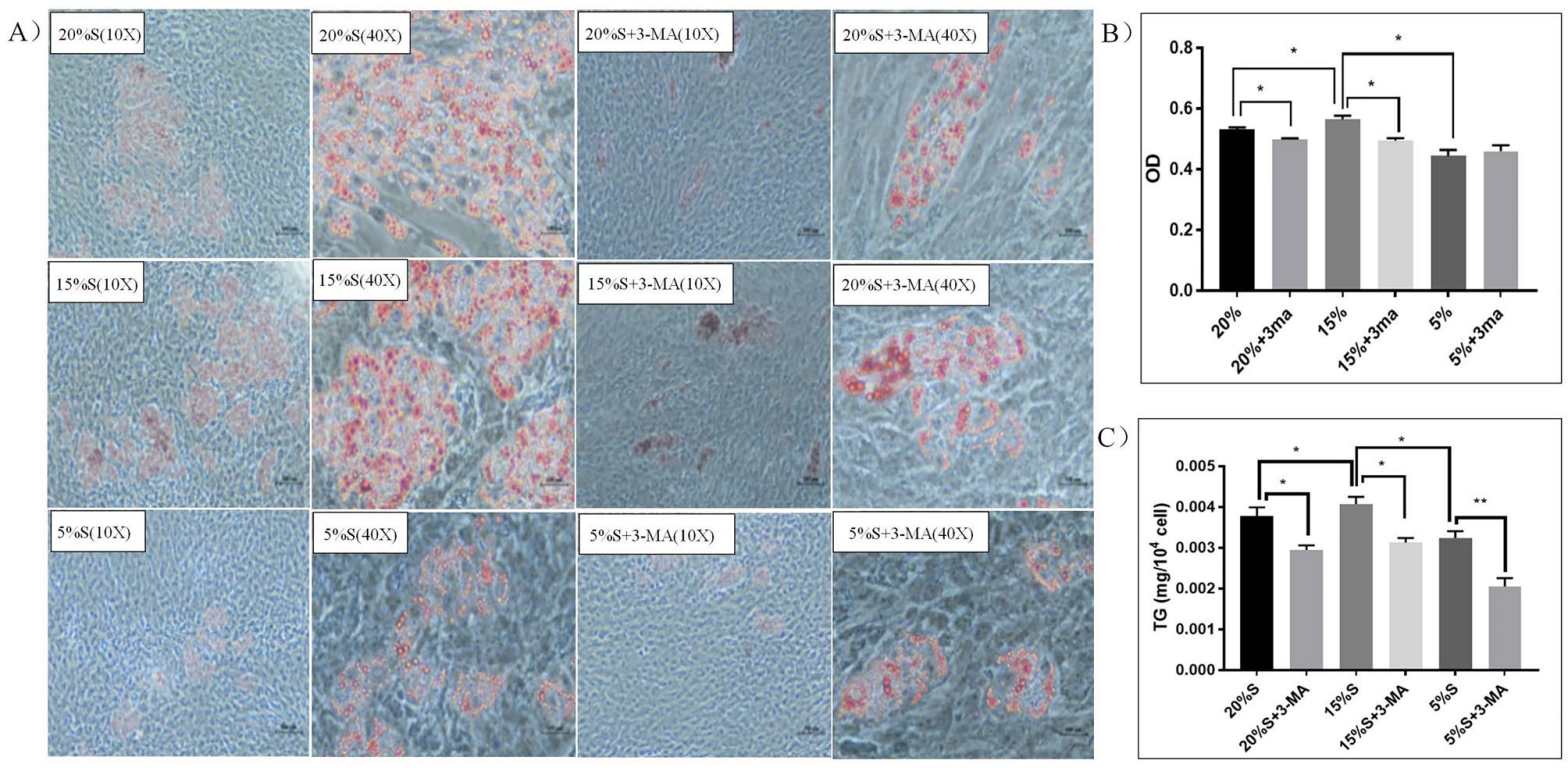
Detection and analysis of fat content and TG levels. (**A**) Detection of fat content by Oil Red O after lipogenic differentiation (10×, 40×); (**B**) Quantitative determination of the OD value after lipogenic differentiation; (**C**) Detection and analysis of TG levels. The data are expressed as the mean ± SD (n = 3) of three experiments. The statistical significance of differences between groups was analyzed using two-tailed Student’s t-tests, and differences were considered statistically significant when *P < 0.05 and **P < 0.01.

After adipogenic differentiation, the lipid and adipocytes in the cells were stained with Oil Red O. The results are shown in Fig. 11A. Lipid droplets dyed orange can be seen in each group. The number of fat drops in the 15% S group was significantly higher than that in the 20% S group and was the lowest in the 5% S group. Compared with the corresponding experimental group, the number of fat drops in the 3-MA groups was significantly reduced (*P* < 0.05). The lipid droplets in cells were quantitatively detected with an Oil Red O detection kit. The results in Fig. 11B show that the OD values at 510 nm of the cells in the 20% S, 20% S + 3-MA, 15% S, 15% S + 3-MA, 5% S, and 5% S + 3-MA groups were 0.533 ± 0.003, 0.499 ± 0.002, 0.569 ± 0.003, 0.495 ± 0.006, 0.443 ± 0.016, and 0.459 ± 0.016, respectively. The quantitative results of cells in the 15% S group were significantly higher than those in the 20% S group and the 5% S group (*P* < 0.05), and the detection results were consistent with the results of Oil Red O staining. There was no significant difference between the groups after adding 3-MA (*P* > 0.05).

#### Detection of TG levels in cells

After adipogenic differentiation, the level of TGs in the cells was quantitatively detected with TG detection kit. The results are shown in Fig. 11C. The TG content in cells of the 20% S, 20% S + 3-MA, 15% S, 15% S + 3-MA, 5% S, and 5% S + 3-MA groups was 0.00375 ± 0.00018, 0.00295 ± 0.00008, 0.00401 ± 0.00014, 0.00313 ± 0.00008, 0.00324 ± 0.00012, and 0.00205 ± 0.00015, respectively. The TG content in the 15% S group was significantly higher than that in the 20% S group and was the lowest in the 5% S group (*P* < 0.05). Compared with the corresponding experimental group, the expression of corresponding TGs decreased significantly in the 3-MA groups (*P* < 0.05).

## Discussion

Autophagy is a protective mechanism that cells use to degrade intracellular contents to maintain nutritional and energy balance dynamics. A certain degree of autophagy helps cells with repair and regeneration, while excessive autophagy has a negative effect on cell function and can lead to cell apoptosis^18^. SMSCs are myogenic stem cells with proliferation and differentiation capabilities after injury or stimulation by certain factors^19^. At present, there is evidence that autophagy can regulate the differentiation of SMSCs^20,21^. However, different degrees of autophagy may have different effects on the metabolism and differentiation functions of cells, which may eventually become an important factor affecting muscle development in vivo. However, related work has not yet been reported^18^. In this study, the serum concentration in the culture medium was controlled to simulate nutrient deficiency and induce autophagy in SMSCs at varying degrees. Autophagy markers, p62 and LC3B, are often used to detect autophagy. LC3B is an autophagy marker, and when autophagy is induced, the cytoplasmic LC3B (i.e., LC3B-I) will cleave off a small peptide and transform into a membrane-type protein (i.e., LC3B-II). An increase in p62 levels usually indicate the inhibition of autophagy^22–24^. In this study, it was found that the expression of p62 in the 15% S group was lower than that in the 20% S group and was the lowest in the 5% S group, while the expression of LC3B-II/ LC3B-I was the opposite. After adding the autophagy inhibitor 3-MA to the cells, the expression of the above autophagy-related proteins was significantly reduced, indicating that the reduction of serum concentration could successfully induce autophagy. The AMPK/mTOR signaling pathway is a classic signaling pathway for autophagy. After AMPK activation, phosphorylation of mTOR-binding protein Raptor will reduce the phosphorylation of mTOR. The activation of AMPK and the inhibition of mTOR cooperate with each other to initiate autophagy^25,26^. In this study, it was found that after the serum concentration was decreased in the SMSC culture system, the p-AMPK/AMPK ratio increased significantly, while the p-mTOR/mTOR ratio decreased significantly, indicating that autophagy regulated by AMPK/mTOR pathway was activated in this process.

Cell metabolism is the response of cells to the external environment for growth, reproduction, and maintenance of structure. The effects of the environment on cell metabolism can be better evaluated by measuring the membrane potential, reactive oxygen species level, apoptosis rate, and ATP levels^27–30^. We found that compared with the 20% S group and the 15% S group, the 5% S group had the highest ATP and ROS levels, the lowest membrane potential, and the highest cell apoptosis rate. The levels of ATP and ATP production and the levels of intracellular ATPase and ATP synthase showed different trends. Intracellular ATPase levels decreased with autophagy, whereas ATP synthase expression increased during autophagy and was highest during mild autophagy. The reasons for these differences need to be further investigated. Compared with the 20% S group, the 15% S group had an increased ROS level, but the changes in membrane potential and cell apoptosis rate were not obvious, indicating that mild autophagy promotes the mitochondrial activity of SMSCs and accelerates cell metabolism but does not increase the percentage of apoptotic cells. Mitochondrial ROS were found to stimulate the PI3K/AKT/mTOR cascade signaling pathway and activate mTORC1 to induce the autophagic signaling pathway and promote muscle differentiation^31^. In this study, we found that different degrees of autophagy occurred and directly affected the AMPK/mTOR pathway, and the production of ROS in cells was also increased, which indirectly suggested that the autophagic pathway may affect skeletal muscle differentiation pathways in multiple ways and that the dominant mechanisms are yet to be further investigated.

Combined with the results of cell transmission electron microscopy and detection of lysosomes in cells, in addition to many autophagic lysosomes, cells in the 5% S group also showed nuclear abnormalities and most mitochondrial structure abnormalities. Autophagic lysosomes were also found in cells of the 15% S group, but the intracellular organelles were relatively intact, indicating that short-term mild autophagy (15% S) promotes the metabolism of SMSCs, while severe autophagy (5% S) causes abnormal cell metabolism and accelerated cell apoptosis. Autophagy and apoptosis are mutually inhibitory. Under normal conditions, autophagy plays a protective role in cells, while apoptosis is responsible for clearing aging or damaged cells, thus maintaining the body at a healthy state^32^. We found that excessive autophagy caused the cell to degrade too many intracellular organelles, leaving the cell unable to survive and pushing it toward apoptosis. Mild cell autophagy, however, promotes cell metabolism, has little effect on cell survival, and does not stimulate cell apoptosis.

SMSCs, a type of stem-like cells with myogenic and lipogenic differentiation capabilities, play an important role in the development and repair of muscle tissues. Autophagy is a key regulatory process in skeletal muscle development, regeneration, and homeostasis, which can activate the differentiation of SMSCs^18,32,33^. Different degrees of autophagy have different effects on cell metabolism, and it may also have different effects on the differentiation function of SMSCs. We investigated the myogenic and lipogenic differentiation of SMSCs. First, in terms of myogenic differentiation detection, we determined the expression of myogenic differentiation marker genes MyoD and MHC. MyoD plays a crucial role in inducing satellite cell differentiation, and the upregulation of MyoD can initiate the myogenic differentiation of SMSCs^34,35^. The proliferation of myoblasts highly expressing MyoD1 ultimately promotes the expression of MHC. MHC can promote the differentiation and fusion of mononuclear cells to form multinucleated muscle fibers, i.e., muscle fibers^36–38^. We found that the protein expression levels of MyoD and MHC in the 15% S group were significantly higher than those in other groups, while the protein expression levels of MyoD and MHC in the 5% S group were significantly lower than those in the 20% S and 15% S groups, indicating that mild autophagy had a significant promotive effect on myogenic differentiation, and severe autophagy showed a significant inhibitory effect on the myogenic differentiation of SMSCs. We then detected the expression levels of lipogenic differentiation marker genes PPARγ and LPL. PPARγ is considered to be the most important regulatory factor for cell lipogenic differentiation, which can promote the differentiation and formation of fat cells^5,6,39^. LPL can hydrolyze lipoproteins in the blood and provide fatty acids necessary for glycerol synthesis, which is positively correlated with the deposition of fat in the muscle. The increase of PPARγ and LPL expression indicated the improvement of cell lipogenic differentiation ability^40^. We found that after the induction of lipogenic differentiation, the expression levels of PPARγ and LPL in the 15% S group were significantly higher than those in the 20% S group, while the expression levels in the 5% S group were the lowest, which molecularly demonstrated that mild autophagy could promote the lipogenic differentiation of SMSCs and that severe autophagy could inhibit the lipogenic differentiation. To further detect the effect of different degrees of autophagy on the lipogenic differentiation ability of SMSCs, Oil Red O was used to stain the lipid substances in each group, and the content of TGs in each group was measured—both of which are the main methods to evaluate the fat content in cells^41,42^. The results showed that the levels of lipid substances and TGs in the 15% S group were the highest, while those in the 5% S group were the lowest, further demonstrating that mild autophagy could promote lipogenic differentiation, while severe autophagy could inhibit it. We followed up on the effects of prolonged serum starvation on SMSCs, and the preliminary results demonstrated that prolonged serum starvation treatment directly inhibited cell metabolism and differentiation after either mild or severe autophagy (data to be published).

Cellular autophagy is a physiological and likely pathological process. We found that autophagy induced by short, mild serum starvation (15% S) is promotive of cellular metabolism and differentiation and presents a physiological promotion of the cellular phenotype. However, more severe serum starvation (5% S) induced severe autophagy, which showed pathological effects on the cells. We followed up with the application of mild autophagy in mice with a “light control diet - normal feeding” recirculating pattern, and our preliminary results showed that the mice in mild autophagy had a significantly higher feed conversion rate and increased muscle production (data to be published). The results suggest that autophagy plays a role in skeletal muscle development and may help design strategies for improving meat production in domestic pigs.

### Supplementary Information

Supplementary Information 1.

Supplementary Information 2.

## Data Availability

All data included in this study are available upon request by contact with the corresponding author.
