# Supplementary material for: Different levels of autophagy induced by transient serum starvation regulate metabolism and differentiation of porcine skeletal muscle satellite cells
Source: Sci Rep. 2023 Aug 12;13:13153. doi: 10.1038/s41598-023-40350-y (PMC10423287; doi:10.1038/s41598-023-40350-y)
Supplement: Supplementary file 1 — Supplementary Information 1. [file 41598_2023_40350_MOESM1_ESM.pdf]

We thank the in-house Editorial Comments. We are not able to provide all images showing full length membranes in figure 1. After gel electrophoresis and membrane transfer, the membrane was cut into several parts and wb hybridization was performed. The blots were cut prior to hybridisation with antibodies. We have included images of all blots in the Supplementary Information file. Images of all gels and blots provided are 300 DPI.

The explanation for the absence of images of adequate length was added in figure 1 legends in the manuscript.

Exposure of MHC and MyoD1 is different due to different blots and different marker strength. Images of MHC and MyoD1 at different exposure times have been provided. After MHC protein detection, the antibody was eluted with stripping buffers and the MyoD1 antibody expression was detected.
