# Supplementary material for: Different levels of autophagy induced by transient serum starvation regulate metabolism and differentiation of porcine skeletal muscle satellite cells
Source: Sci Rep. 2023 Aug 12;13:13153. doi: 10.1038/s41598-023-40350-y (PMC10423287; doi:10.1038/s41598-023-40350-y)

Fig.A-LC3B

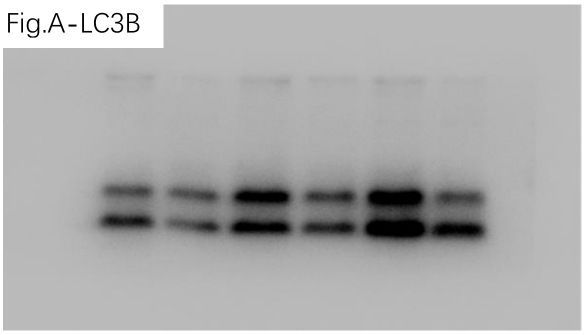

Fig.B-p62

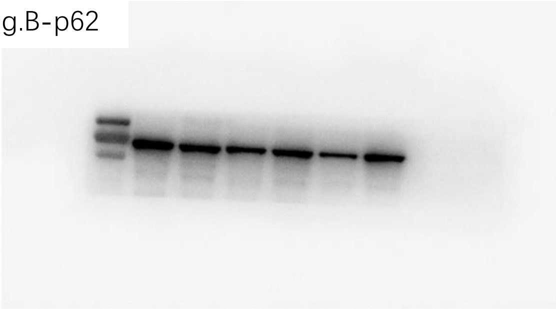

Fig.C-Tubulin

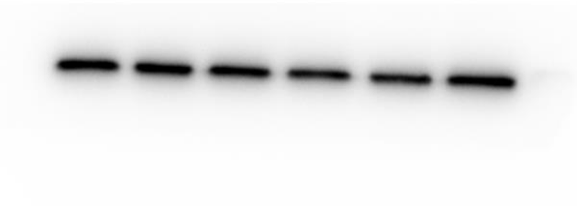

Fig.D-p-mTOR

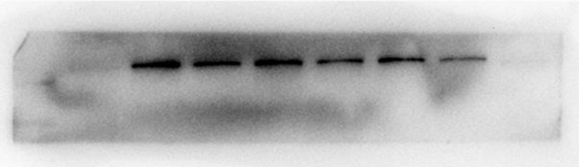

Fig.E-mTOR

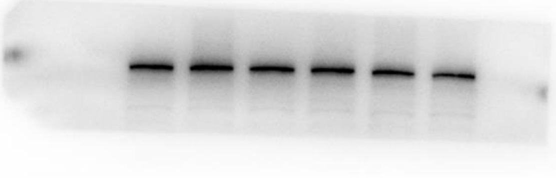

Fig.F-p-AMPK

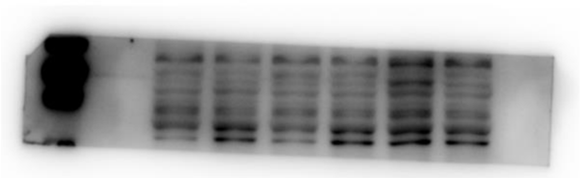

Fig.G-AMPK

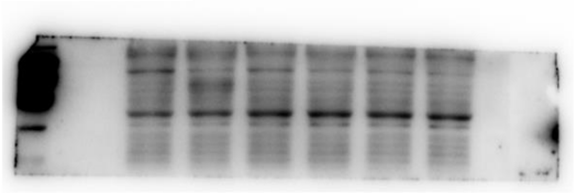

Fig.H-Tubulin

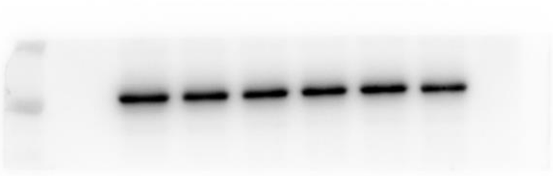

Fig.I-MHC

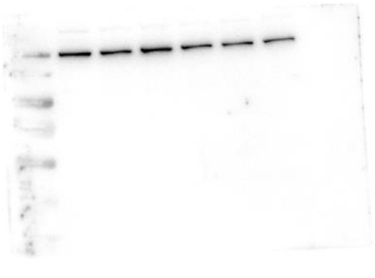

Fig.J-MyoD1

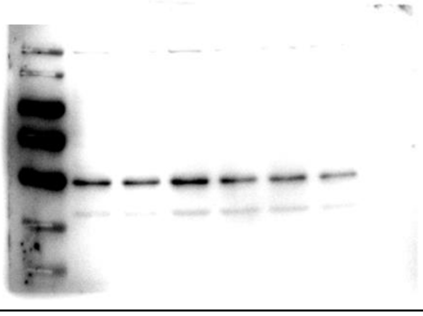

Fig.K-MHC and MyoD1 of different exposure time

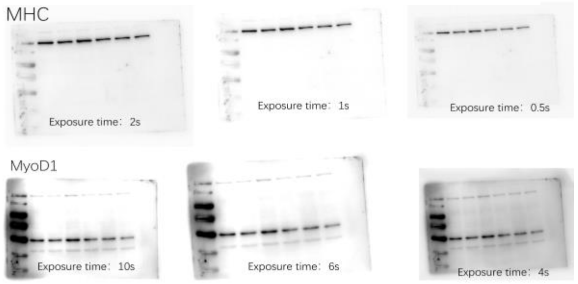

Fig.L-Ponceau（black-and white graph）

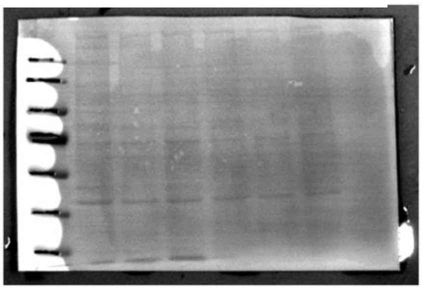

Fig.M-Ponceau（Color graph ）

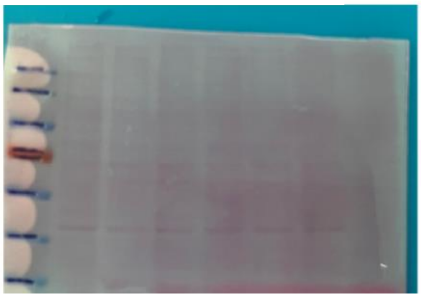

Supplement: Supplementary file 2 — Supplementary Information 2. [file 41598_2023_40350_MOESM2_ESM.pdf]
